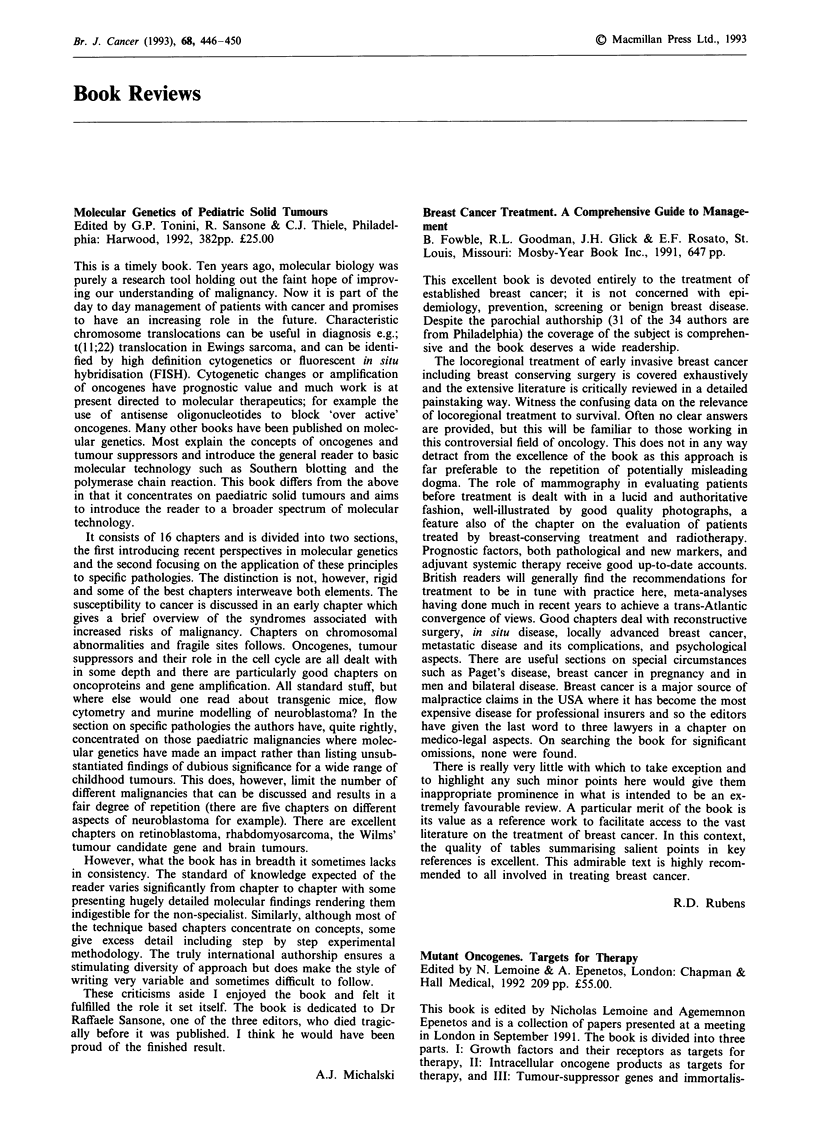# Molecular Genetics of Pediatric Solid Tumours

**Published:** 1993-08

**Authors:** A.J. Michalski


Br. J. Cancer (1993), 68, 446-450 © Macmillan Press Ltd., 1993

### Book Reviews

**Molecular Genetics of Pediatric Solid Tumours**
Edited by G.P. Tonini, R. Sansone & C.J. Thiele, Philadelphia: Harwood, 1992, 382pp. £25.00

This is a timely book. Ten years ago, molecular biology was purely a research tool holding out the faint hope of improving our understanding of malignancy. Now it is part of the day to day management of patients with cancer and promises to have an increasing role in the future. Characteristic chromosome translocations can be useful in diagnosis e.g.; t(11;22) translocation in Ewings sarcoma, and can be identified by high definition cytogenetics or fluorescent *in situ* hybridisation (FISH). Cytogenetic changes or amplification of oncogenes have prognostic value and much work is at present directed to molecular therapeutics; for example the use of antisense oligonucleotides to block 'over active' oncogenes. Many other books have been published on molecular genetics. Most explain the concepts of oncogenes and tumour suppressors and introduce the general reader to basic molecular technology such as Southern blotting and the polymerase chain reaction. This book differs from the above in that it concentrates on paediatric solid tumours and aims to introduce the reader to a broader spectrum of molecular technology.

It consists of 16 chapters and is divided into two sections, the first introducing recent perspectives in molecular genetics and the second focusing on the application of these principles to specific pathologies. The distinction is not, however, rigid and some of the best chapters interweave both elements. The susceptibility to cancer is discussed in an early chapter which gives a brief overview of the syndromes associated with increased risks of malignancy. Chapters on chromosomal abnormalities and fragile sites follows. Oncogenes, tumour suppressors and their role in the cell cycle are all dealt with in some depth and there are particularly good chapters on oncoproteins and gene amplification. All standard stuff, but where else would one read about transgenic mice, flow cytometry and murine modelling of neuroblastoma? In the section on specific pathologies the authors have, quite rightly, concentrated on those paediatric malignancies where molecular genetics have made an impact rather than listing unsubstantiated findings of dubious significance for a wide range of childhood tumours. This does, however, limit the number of different malignancies that can be discussed and results in a fair degree of repetition (there are five chapters on different aspects of neuroblastoma for example). There are excellent chapters on retinoblastoma, rhabdomyosarcoma, the Wilms' tumour candidate gene and brain tumours.

However, what the book has in breadth it sometimes lacks in consistency. The standard of knowledge expected of the reader varies significantly from chapter to chapter with some presenting hugely detailed molecular findings rendering them indigestible for the non-specialist. Similarly, although most of the technique based chapters concentrate on concepts, some give excess detail including step by step experimental methodology. The truly international authorship ensures a stimulating diversity of approach but does make the style of writing very variable and sometimes difficult to follow.

These criticisms aside I enjoyed the book and felt it fulfilled the role it set itself. The book is dedicated to Dr Raffaele Sansone, one of the three editors, who died tragically before it was published. I think he would have been proud of the finished result.

A.J. Michalski